# Airway Management of an Infant With Giant Neck Macro-Cystic Hygroma Utilizing a High-Flow Nasal Cannula

**DOI:** 10.7759/cureus.46865

**Published:** 2023-10-11

**Authors:** Shiwei Huang, Zhihao Wang, Yauwai Chan, Tao Jiang

**Affiliations:** 1 Anesthesiology, The University of Hong Kong-Shenzhen Hospital, Shenzhen, CHN

**Keywords:** pediatric anesthesia, fiber-optic bronchoscope, laryngeal spasm, high-flow nasal cannula, difficult airway management, macro-cystic hygroma

## Abstract

Background: Cystic hygroma is a congenital lymphatic malformation. It may present as a huge mass in the neck, jeopardizing airway patency and prolonging intubation time, resulting in hypoventilation and hypoxemia. We used a high-flow nasal cannula to decrease the risk of hypoxemia and provide anesthesiologists sufficient time to perform tracheal intubation in young infants.

Case presentation: A 33-day-old infant (height, 55 cm; weight, 5.05 kg) was diagnosed with macro-cystic hygroma of the right neck. Considering the progressive enlargement of the macrocystic hygroma and its impact on the airway, urgent intervention becomes imperative. Among the available treatment modalities, percutaneous cyst aspiration and sclerotherapy performed under ultrasound guidance represent the most commonly chosen approach. During the induction of general anesthesia, the otolaryngologists were on standby and prepared for emergency tracheotomy. The anesthesiologists chose total intravenous anesthesia induction while maintaining spontaneous breathing. A high-flow nasal cannula was used to keep the infant oxygenated, and endotracheal intubation was successfully performed using a C-MAC video laryngoscope and fiber-optic bronchoscope.

Conclusions: Airway management is the biggest challenge for anesthesiologists when delivering general anesthesia to infants with neck macro-cystic hygroma. Total intravenous anesthesia could be a choice for induction without considering compromised respiration and the side effects of inhalational anesthetics. A high-flow nasal cannula can be used in young infants to maintain oxygenation and allow anesthesiologists a longer time to perform intubation.

## Introduction

Cystic hygroma, a benign congenital neoplasm that mainly presents as a soft-tissue mass, and is also called a lymphatic malformation, is an anomaly of the lymphatic system characterized by single or multiple cysts within the soft tissue. In most cases of cystic hygromas, 70-80% involve the neck and lower part of the face; other sites are the axilla, superior mediastinum, retroperitoneum, mesentery pelvis, and lower limbs. Approximately 50-60% of these malformations appear before one year of life and 80-90% before the end of the second year of life [1]. Fluid accumulation in the dilated lymphatic and surrounding connective tissues leads to progressive lymphedema, and nonimmune hydrops often occur [2]. Cystic hygroma of the neck region often presents challenges to anesthesiologists because of the extension of the neck and airway. High-flow nasal oxygenation (HFNO) has proven to be highly effective for patients admitted to the intensive care unit (ICU), operating room for deep sedation, or post-anesthetic care unit after surgery [3-5]. It also shows superiority with a significantly lower risk of desaturation and upper airway obstruction and fewer airway maneuvers than conventional nasal cannulas [4]. It has not been widely used in very young infants during surgery or anesthesia induction. Safety has been guaranteed since HFNO has been widely used in young infants for acute viral bronchiolitis as an initial respiratory treatment in the ICU [6,7]. We aimed to introduce this new technique to patients who are at higher risk of desaturation and in whom a longer desaturation time may be required with the expected difficult airway management. 

## Case presentation

**Figure 1:**
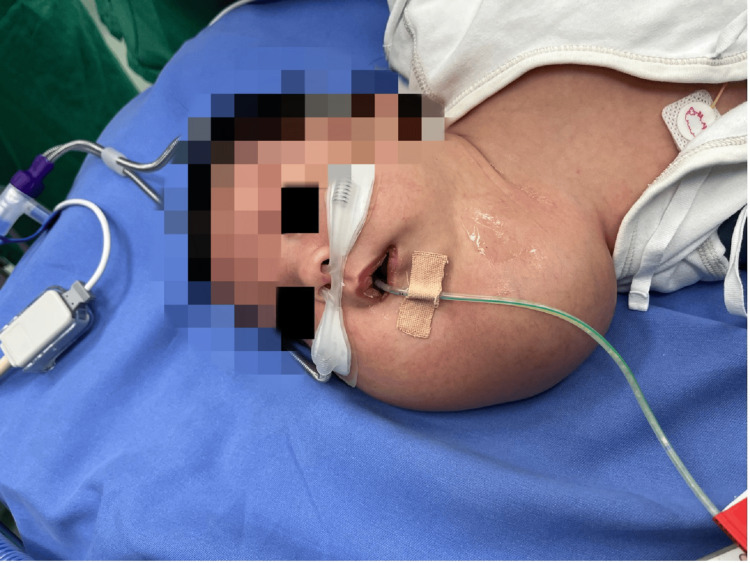
The patient with massive swelling on the right side of his neck

A 33-day-old full-term Chinese male infant weighing 5.05 kg presented with massive swelling on the right side of his neck, restricted mouth opening, and difficulty feeding (Figure 1).

The postnatal diagnoses included the following: (1) water-cystic lymphangioma of the right neck; (2) neonatal patent ductus arteriosus; and (3) neonatal hyperbilirubinemia.

**Figure 2:**
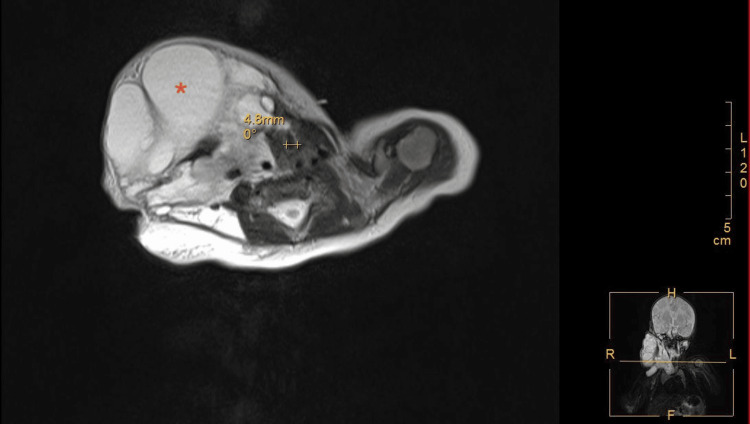
Magnetic resonance imaging of the macro-cystic hygroma（*） and the narrowed trachea（++） as measured

The planned surgery included percutaneous cyst aspiration and sclerotherapy under ultrasound guidance. The general conditions of this infant before surgery were as follows: spontaneous breathing, 30-40 beats per minute (bpm); peripheral oxygen saturation (SpO2) on room air, 90%-98%; heart rate, 140-165 bpm; and systolic pressure, 45-68 mmHg. Upon pulmonary auscultation, breath sounds were rough, wheezing sounds could be heard, and slight suprasternal and supraclavicular retractions could be observed during crying. Cardiac auscultation revealed no murmur. Laboratory tests revealed normal complete blood count and liver, kidney, and coagulation functions. Magnetic resonance imaging 1.5T with contrast was performed on day 28 (Figure 2).

An extensive multilocular cystic mass was noted in the right maxillofacial neck and upper-chest mediastinum, with a size of approximately 8.5 × 7.5 × 10.9 cm. Worrisome for lymphangioma with infection or bleeding. The trachea deviated to the left and narrowed under compression. The diameter of the cricoid cartilage was approximately 4.6 mm, as measured using ultrasound.

The anesthesia plan included general anesthesia with endotracheal intubation. Airway management included three methods, plans A, B, and C.

Under plan A, spontaneous breathing was maintained with HFNO; esketamine and propofol were selected as intravenous anesthetics. Considering the severe upper airway anomalies, the infant’s own respiratory effort would be essential for maintaining an open airway. Attempted intubation without muscle relaxants was preferred [4,7]. Once we achieved deep sedation, we placed the infant in a supine position with the head and neck tilted to the affected side, and the glottis was exposed using a C-MAC video laryngoscope (0# blade, 8402ZX; STORZ, Germany). Using video-enhanced visualization of the larynx, 2% lidocaine 1ml was sprayed into the throat and trachea. Moreover, endotracheal intubation (3.0 mm, 9336E; COVIDIEN, Thailand) was performed using the video laryngoscope combined with the fiber-optic bronchoscope (2.2 mm, H110266; PENTAX, Japan) after adequate preoxygenation.

Under plan B, after inserting an intubating laryngeal mask airway (1.0# air-Q; Cookgas, Malaysia), endotracheal intubation was performed through the intubating laryngeal airway using a fiber-optic bronchoscope.

Under plan C, if intubation failed, a face mask or laryngeal mask airway would be used for emergency ventilation. Otolaryngologists could perform a tracheotomy or awaken the baby.

**Figure 3:**
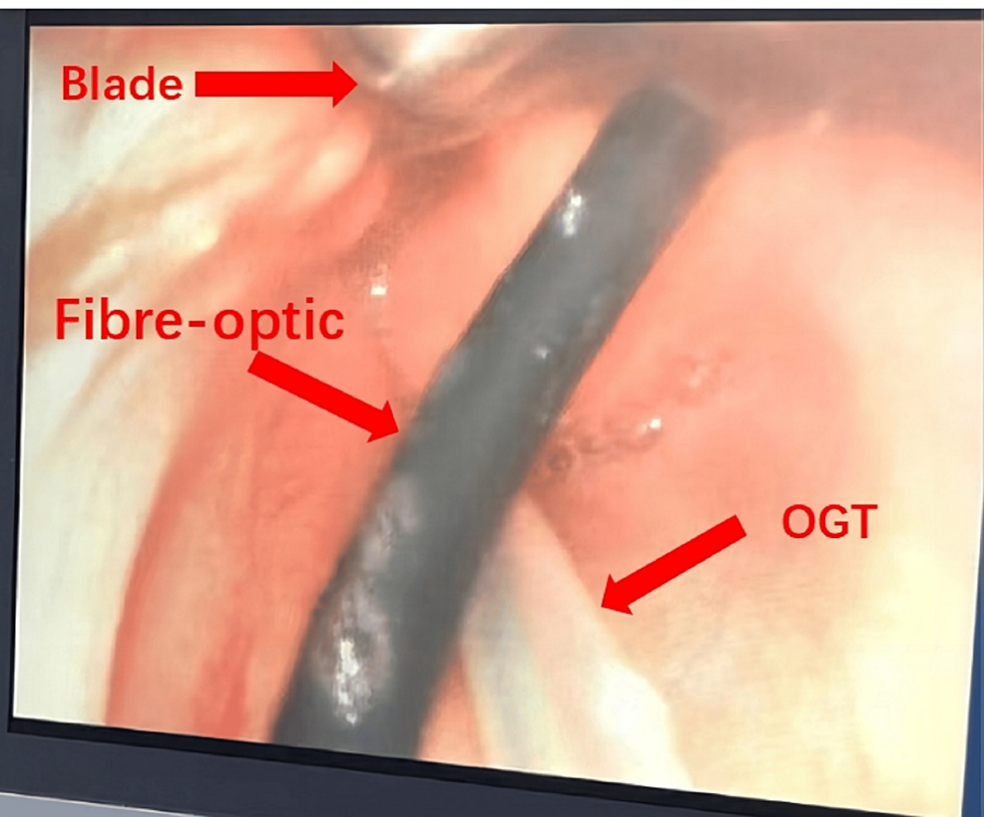
The perspective observed through the C-MAC videoscope is illustrated with a red arrow pointing towards the videoscope blade, fiber-optic endoscope, and oral gastric tube (OGT)

We used HFNO (OPT318/RT330; Maurice Paykel, New Zealand) (initial flow rate, 1.5 L/kg/min; FiO2, 100%). Anesthesia was induced using esketamine and propofol. Endotracheal intubation was performed while maintaining the infant’s spontaneous breathing under total intravenous anesthesia in the supine position with an oral gastric tube in situ. Rescue tracheostomy was performed by the surgeon during induction. Anesthesia was achieved using intermittent boluses of esketamine 15 mg and propofol 10 mg. Our initial attempt at intubation using a videoscope yielded limited visibility of the glottis because of the obstructed view of the vocal cords. Lidocaine was administered to the glottis to enhance hemodynamic stability. However, this approach elicited laryngeal spasm, which resulted in a transient decrease in the infant’s SpO2 to 80% that lasted approximately 10 s. Without delay, we ceased the procedure by retracting the blade and promptly administered 100% oxygen via a face mask. The laryngeal spasm subsided spontaneously, and SpO2 gradually improved to 100%. Owing to the challenging vocal cord visualization and increased risk of tissue damage, bleeding, and swelling associated with blind intubation, we opted to employ a combination of videoscopy and fiber-optic bronchoscopy for the second intubation attempt. Figure 3 provides a visual representation of our second intubation attempt as observed through videoscopy. It displays the layout of the bronchoscopy, the oral gastric tube (OGT), and the intubation blade.

Endotracheal intubation was successfully performed under the guidance of a fiber-optic bronchoscope without desaturation. The endotracheal tube (ETT) used was 3.0# uncuff. Anesthesia was maintained using fentanyl, sevoflurane, and cis-atracurium. The entire surgical procedure was almost 1 hour and uneventful. After communicating with surgeons and neonatologists, considering postoperative bleeding and edema endangering the airway, the patient was returned to the NICU while intubated. The ETT was successfully withdrawn 2 days after surgery.

## Discussion

Lymphangioma, or cystic hygroma, is a congenital malformation of the lymphatic system that is mainly present at birth [2]. The head and neck regions are the most frequently affected sites [8]. Huge neck cystic hygroma compresses the airway and mouth of the patient, resulting in difficult lactation and dyspnoea. Anesthesiologists face significant challenges if such patients require surgical treatment. The main difficulty associated with general anesthesia is airway management. Premedication is avoided because these patients are susceptible to apnoea due to compromised airways. After topical airway anesthesia, awake intubation under the guidance of a fiber-optic bronchoscope is also an option [9]. Nevertheless, given the challenges posed by the infant's limited ability to cooperate effectively and the potential for the patient's agitation to result in intracapsular hemorrhage and exacerbation of airway compression, this approach was not employed. Ultimately, intubation was supposed to be carried out under general anesthesia or deep sedation, and spontaneous breath should be maintained until intubation succeeded during the procedure [7,10]. After induction of the anesthesia, oxygenation is usually provided by intermittent positive-pressure ventilation via a facemask. However, with an expected difficult airway, especially when a fiber-optic bronchoscope is required, the risk of hypoxemia increases. Owing to the higher oxygen requirement, decreased functional residual capacity, and higher risk of airway collapse, the risk of rapid desaturation is higher in children [11,12]. In a previous study, the mean desaturation times at which SpO2 reached 90% were 160, 382, and 97 s in children, adolescents, and infants, respectively. HFNO extended the safe apnoea time in infants (0-6 months) from 109.2 to 192 s [13-14]. To allow anesthesiologists a longer time to perform intubation with a fiber-optic bronchoscope, a body weight-tailored (1 L/kg) HFNO was applied.

The final airway management plan included the following: (1) a high-flow humidification oxygen inhalation device supplied adequate oxygen to the patients with spontaneous breathing, and a T-piece pipeline was prepared to provide emergency positive-pressure ventilation; (2) esketamine did not affect the patient’s breathing and reached the appropriate depth of anesthesia for visual laryngoscopy, with low-dose propofol used to reduce the incidence of laryngeal spasms without affecting breathing, and muscle relaxants were avoided; (3) a C-MAC visual laryngoscope was chosen for intubation, but, when attempts failed, a C-MAC visual laryngoscope was used to facilitate fiber-optic laryngoscopy [15]; (4) lidocaine was sprayed into the glottis and trachea to effectively reduce airway stimulation during laryngoscopy and tracheal intubation; (5) otolaryngologists were present, and if necessary, emergency aspiration of cystic fluid was to be carried out to reduce airway compression, as well as endotracheal intubation, and in the event ventilation or intubation was not possible, otolaryngologists would perform an emergency tracheotomy.

Prior to this case, there had been few reports on the use of HFNO in very young infants during surgery or anesthesia induction [16-17]. We used this strategy to decrease the incidence of hypoxia and offer anesthesiologists adequate time to perform fiber-optic laryngoscopy for a difficult airway. No hypoxic events occurred during the fiber-optic laryngoscope manipulation. The anesthesiologist felt that the time was prolonged enough for fiber-optic laryngoscope-facilitated intubation, which is not commonly observed in difficult airways.

## Conclusions

Airway management is particularly challenging when delivering general anesthesia to infants with neck macro-cystic hygroma. Total intravenous anesthesia can be a choice for induction without considering compromised respiration and the side effects of inhalational anesthetics. A high-flow nasal cannula can be used in young infants to maintain oxygenation and allow anesthesiologists a longer time to perform intubation. Front-of-neck access is indicated in the "cannot intubate, cannot oxygenate" scenario when an ENT surgeon is available. Tracheotomy is generally viewed as the last option; however, it cannot be disregarded. In terms of preserving life, all potential options should be pursued and deemed valuable. In certain case studies, tracheotomy is also being considered. 
